# Two-Stage Fatigue Life Decoupling and Reflective Crack Retardation Mechanisms of Interlayer Systems in Rigid–Flexible Composite Beams

**DOI:** 10.3390/ma19204347

**Published:** 2026-10-15

**Authors:** Rui Sun, Lingyun Kong

**Affiliations:** School of Civil Engineering, Chongqing Jiaotong University, Chongqing 400074, China

**Keywords:** three-point bending test, crack-resistant interfacial layer, reflective cracking, two-stage fatigue life decoupling, cumulative dissipated energy

## Abstract

For rigid-flexible composite pavement structures, traditional macroscopic assessment of crack-resistant interlayers is typically based on total fatigue life alone, without differentiating between crack propagation through the rigid substrate and crack arrest behavior at the modulus-mismatched interface. To fill this research gap, the present work characterizes the stage-specific crack retardation mechanisms of three distinct interlayer systems. Pre-notched composite beam specimens with dimensions of 300 mm × 100 mm × 100 mm were fabricated, each composed of a 50 mm C40 concrete base layer, a spray-applied interlayer coating (epoxy emulsified asphalt [EEA], SBS-modified asphalt, or rubberized asphalt), and a 50 mm SMA-13 asphalt mixture overlay. Static and cyclic three-point bending tests were conducted on these specimens across a range of test conditions: temperatures from −10 °C to 45 °C, loading frequencies of 1–15 Hz, and stress levels of 0.3–0.5. Using a combined approach of load–deflection hysteretic energy dissipation analysis and a cyclic J-integral (∆J) modified Paris law, the total fatigue life was resolved into two separate stages: crack propagation in the concrete base (N_c_) and crack propagation across the interface into the overlay (N_a_). The test results identify optimal interlayer binder dosages of 0.8 kg/m^2^ for both EEA and SBS-modified asphalt, and 2.0 kg/m^2^ for rubberized asphalt. EEA exhibited the highest peak static load capacity of 12.6 kN, but was followed by abrupt brittle fracture once the peak load was exceeded. Rubberized asphalt, by contrast, retained full structural integrity even at large mid-span deflections up to 7 mm. According to the two-stage fatigue life analysis, the crack propagation life in the concrete substrate (N_c_ ≈ 35,000 cycles) is entirely unaffected by the type of interlayer material. All divergence in anti-cracking performance occurs after the crack reaches the interface: rubberized asphalt prolonged N_a_ to more than 120,000 cycles, with a total cumulative dissipated energy of roughly 6000 J. These findings verify that flexible interlayer coatings suppress reflective cracks mainly via large-deformation deformation coordination and hysteretic energy dissipation at the post-interface stage, instead of altering the fracture behavior of the underlying rigid base layer.

## 1. Introduction

Rigid-flexible composite pavements, typically composed of a cement concrete base course overlain by an asphalt surface layer, integrate high load-bearing capacity with favorable riding quality, and have been widely adopted in highway and long-tunnel pavement engineering [1]. However, under long-term service conditions, the combined action of repeated heavy traffic loads and cyclic temperature variations drives the upward propagation of base joints and pre-existing microcracks into the asphalt surface layer, eventually forming full-depth cracks [2,3]. When vehicle loads pass across base joints, instantaneous vertical differential settlement and horizontal opening deformation occur between adjacent slabs. This subjects the layer interface and overlying asphalt surface layer to a long-term coupled stress state combining Mode I (opening) and Mode II (in-plane shear) fracture modes [4,5]. Once reflective cracks penetrate the pavement surface, water ingress through the cracks triggers a cascade of distresses including interface delamination and base course pumping, which significantly reduces the pavement’s service life [6]. Placing a crack-resistant interfacial layer (also referred to as a stress-absorbing layer) between structural layers is a widely used engineering measure to suppress reflective crack initiation and propagation [7].

Existing crack-resistant interlayer coatings can be divided into two distinct categories based on their structural and mechanical properties. The first group is rigid thermosetting binders, with epoxy emulsified asphalt (EEA) as a typical representative. Such materials form dense cross-linked structures after curing, which confer high interlayer bond strength and shear modulus. While cured epoxy binders effectively limit interlayer relative slip under high load conditions, their low deformation tolerance renders them prone to brittle fracture when subjected to large slab differential displacements [8]. Dai et al. [9] and Kong et al. [8] performed comparative studies via direct shear and tensile tests, respectively. Their results show that cured epoxy resin systems can achieve an interfacial ultimate shear strength of up to 2.0 MPa, which effectively restricts interlayer relative slip in the early service stage under heavy loads. Nevertheless, these materials have limited deformation tolerance and are prone to brittle fracture under large strains induced by sudden temperature drops or substantial slab differential movement [10]. The second group is flexible elastomeric binders, typically represented by SBS-modified asphalt and rubberized asphalt. These materials primarily dissipate strain energy through viscoelastic deformation mechanisms [11]. Li et al. [7] assessed the crack resistance of asphalt rubber stress-absorbing membrane interlayers (SAMI) via a suite of laboratory tests, including small beam bending tests at −10 °C and 15 °C, low-temperature semicircular bending (SCB) tests, crack propagation SCB tests, and overlay tests (OT), and further explored how gradation parameters affect interlayer performance. Pan et al. [12] combined thermal stress restrained specimen tests (TSRST) with finite element analysis to characterize the cracking resistance of warm-mix rubberized asphalt mixtures (WMRMA). Their results indicated that even with a 30 °C reduction in mixing and compaction temperatures, WMRMA still maintained favorable low-temperature performance, which could delay the initiation and propagation of reflective cracks in asphalt pavements and prolong pavement service life. Xu et al. [13] examined the effects of aging on the viscosity and tensile properties of SBS-modified asphalt using viscosity tests, force ductility tests, and molecular dynamics simulations. Yu et al. [14] also conducted related research on the aging behavior of SBS-modified asphalt. It has been demonstrated that aging alters the colloidal structure of asphalt binder and degrades its properties; the network structure formed by SBS modifiers is also destroyed, leading to remarkable changes in rheological properties and further impairing the service performance and durability of SBS-modified asphalt pavements [15]. Existing research on these anti-cracking interlayer coatings has mostly focused on macroscopic performance metrics, including direct shear strength and total fatigue life (N_f_). This approach treats the entire composite pavement structure as a single lumped system, however, and thus cannot address a key mechanistic question: whether the thin interlayer coating physically impedes upward crack growth inside the underlying concrete slab, or whether its anti-cracking benefit acts solely at the bimaterial interface and within the overlying asphalt layer. In addition, the application of classical linear-elastic fracture mechanics (LEFM) to reflective cracking analysis has notable theoretical limitations. The conventional stress intensity factor (∆K) is derived for homogeneous isotropic materials. When a crack propagates from a stiff concrete base into a compliant viscoelastic asphalt interlayer, the substantial mismatch in elastic modulus and the accompanying extensive plastic deformations mean that the core assumptions of LEFM no longer hold.

To fill these knowledge gaps, a mechanistic hypothesis is put forward in this work: the thin interlayer coating imposes no structural restriction on the kinetics of crack propagation within the rigid concrete substrate, and its crack retardation effect is triggered only when the crack reaches the interface, controlled by deformation compatibility and hysteretic energy dissipation. This hypothesis was verified via three-point bending tests on pre-notched rigid-flexible composite beams. The bottom-notch setup produces a predominant global Mode I (opening) stress field, while the abrupt modulus difference at the concrete–asphalt interface generates local in-plane shear stresses, closely reproducing the mixed-mode (Mode I + Mode II) fracture conditions caused by moving traffic loads. A Paris law formulation corrected with a cyclic J-integral (∆J) was adopted to account for heterogeneous yielding, enabling the total fatigue life to be partitioned into two distinct spatial-temporal phases: base course crack propagation (N_c_) and interface–overlay crack propagation (N_a_). This two-stage partitioning approach offers a reliable fracture-mechanics framework for understanding the fatigue performance of various interlayer systems.

## 2. Specimen Preparation and Three-Point Bending Test

### 2.1. Test Materials

In composite pavement structures located in heavy-traffic highway tunnels or rehabilitated from existing rigid pavements, the bottom PCC slab serves as the core load-carrying component. According to the Chinese highway design specifications JTG D40-2011 [16], PCC base courses designed for heavy to extra-heavy traffic levels require a minimum 28-day flexural strength (f_r_) of 5.0 MPa, a performance threshold that aligns with concrete strength grades C35 through C40. Given this requirement, the rigid substrate in this study was cast using C40-grade ordinary Portland cement concrete. The concrete was mixed with grade P·O 42.5R early-strength ordinary Portland cement (compliant with Chinese national standards), river sand, crushed stone, and tap water at a mass ratio of 1:1.42:2.76:0.41. Three different bituminous binders were chosen for the sprayed interlayer coatings in this study, and their basic material properties are described below to explain their distinct fracture behaviors. The EEA binder is a thermosetting modified asphalt prepared from No. 70 base asphalt, bisphenol A epoxy resin (E-51) and a water-based polyamide curing agent. After curing, it develops a rigid three-dimensional cross-linked network structure, which endows the binder with high cohesive strength but very limited ductility after yielding, supplied by Guangzhou Jiabaili Building Materials Co., Ltd. (Guangzhou, China). For the SBS-modified asphalt binder, the raw materials include No. 70 base asphalt, linear styrene-butadiene-styrene (SBS) block copolymer and a sulfur-based crosslinking stabilizer. The SBS polymer phase forms a physical elastomeric network in the asphalt matrix, giving the binder a well-balanced elastoplastic mechanical response, sourced from Guangzhou Wanqiao Waterproof & Anticorrosive Coatings Co., Ltd. (Guangzhou, China). The rubberized asphalt binder is produced by blending No. 70 base asphalt with recycled crumb rubber. In the blending process, the crumb rubber particles absorb light oil fractions in the asphalt and swell, forming a highly viscous suspension system. Strain energy is dissipated in this system mainly through extensive viscoelastic flow and internal friction between rubber particles and the asphalt phase, provided by China Railway Weiye Waterproof Materials Technology Co., Ltd. (Jinan, China). The flexible surface layer was formed with an SMA-13 stone matrix asphalt mixture, supplied by Jinan Jiaodi New Materials Co., Ltd. (Jinan, China).The mixture proportion was designed and optimized to satisfy volumetric design targets, including an air void content of 4.1%, a voids in mineral aggregate of 16.2% and a voids filled with asphalt of 74.8%. The resulting compacted mixture exhibited a bulk specific gravity of 2.46 g/cm^3^, with an optimum asphalt content of 5.8% by mass of the total mixture. Crushed limestone was adopted as the coarse aggregate, with a nominal maximum particle size of 13.2 mm, and limestone powder was used as the mineral filler; both materials were obtained from Xinlei Mineral Powder Factory (Shijiazhuang, China). The physical and mechanical properties of all raw materials meet the requirements of Chinese highway industry standards JTG F40-2004 [17] and JTG E42-2005 [18].

### 2.2. Specimen Preparation and Concrete Strength Verification

At on-site construction locations, ambient temperature fluctuations and rainfall during concrete mixing, transport and placement inevitably affect the free water content, slump retention and cement hydration rate of fresh concrete. To exclude the influence of such weather factors in this laboratory study, all concrete mixing and casting procedures were conducted indoors under constant temperature and humidity control. After pouring at 20 ± 2 °C, the prepared concrete specimens (150 mm × 100 mm × 50 mm) and six standard cube specimens (150 mm × 150 mm × 150 mm) were covered with polyethylene film. At 24 h after demoulding, all specimens were transferred to a standard curing chamber (20 ± 2 °C, relative humidity ≥ 95%) for 28 days of standard curing in accordance with JTG 3420-2020 [19]. Uniaxial compression and flexural tests were performed on specimens cured for 28 days. The cubic compressive strength ranged from 44.6 MPa to 48.5 MPa with a standard deviation of 1.42 MPa, and the average flexural strength reached 5.4 MPa. These results confirm that the test concrete meets the target C40 strength grade, with a typical strength surplus above the design value, which is characteristic of structural concrete mixes.

The top surface of each dried concrete slab was sandblasted to achieve a mean texture depth of 0.6 ± 0.1 mm, a value consistent with standard field milling requirements for rigid-flexible composite overlays as defined in JTG/T 3364-02-2019 [20]. The volumetric sand patch method was adopted to quantitatively verify the uniformity of surface texture across all slabs. Five gradient application rates were designed for each coating material to determine the optimal spray dosage. For EEA and SBS-modified asphalt, the rates ranged from 0.4 to 1.2 kg/m^2^ at 0.2 increments. The rate range for rubberized asphalt was shifted upward to 1.6 to 2.4 kg/m^2^ in the same increment, to account for rubber particle swelling and the higher zero-shear viscosity of this binder. A two-stage curing schedule was applied to EEA-coated specimens. Specimens were first held at 20 °C for 24 h to complete emulsion breaking, and then cured at 60 °C for 48 h to achieve full epoxy crosslinking. Both SBS-modified asphalt and rubberized asphalt were hot-applied and allowed to cool naturally to room temperature. After all mechanical tests, actual interlayer thickness values were measured from sawn cross-sections using a 50× digital optical microscope. At the optimal application rates of 0.8 kg/m^2^ for EEA and SBS, and 2.0 kg/m^2^ for rubberized asphalt, the measured thickness values were 0.85 mm, 0.92 mm and 2.15 mm respectively, with spatial deviation kept below ±0.15 mm along the entire beam length.

After interlayer coating preparation, the SMA-13 asphalt mixture was placed and compacted on the coated concrete substrate to a nominal thickness of 50 mm. Compaction was performed using a static press at 140–160 °C with a constant loading rate of 1 mm/min. The composite slabs were demoulded and subjected to 48 h of thermal conditioning at 60 °C, before being sawn into individual beam specimens with dimensions of 300 mm × 100 mm × 100 mm. A single central notch was machined into the bottom surface of the concrete base layer using a CNC diamond wire saw. Notch width was controlled with high precision to 2.0 ± 0.1 mm. Three different initial notch depths (a_0_) of 10, 15 and 20 mm were prepared, with a vertical machining tolerance of ±0.5 mm. These corresponded to initial notch-to-height ratios (a_0_/H) of 0.1, 0.15 and 0.2 respectively. Four replicate specimens were manufactured for each combination of test variables.

### 2.3. Three-Point Bending Test

Over the service life of composite tunnel pavements, base course joints and pre-existing microcracks are exposed to mixed-mode fracture conditions combining Mode I (opening) and Mode II (in-plane shear). Under symmetric loading, the initial crack at the bottom notch of the test beam develops under a predominantly Mode I stress field. As the crack extends toward the heterogeneous interlayer interface, the large elastic modulus mismatch between rigid concrete and flexible asphalt drives local deformation incompatibility, which in turn generates a Mode II shear component at the interface. This laboratory test setup has intrinsic limitations. The simply supported beam configuration does not reproduce the Winkler elastic foundation support and lateral restraint that exist in real pavement systems, and static cyclic loading fails to fully capture the principal stress rotation caused by moving traffic loads. To address this, the present work uses the macroscopic cyclic J-integral (∆J) derived from load–deflection hysteresis loops. This energy-based approach combines Mode I opening and Mode II sliding contributions into a single equivalent fracture driving force, allowing objective performance comparison of different coating materials [21].

#### 2.3.1. Static Three-Point Bending Test

Prior to testing, the width and height of each specimen were measured using a vernier caliper (Guilin Guanglu Measuring Instrument Co., Ltd., Guilin, China) with an accuracy of 0.1 mm. For each composite beam, the cross-sectional width (b) and height (H) were measured at three positions along the specimen length, and average values were adopted to minimize dimensional deviations introduced during specimen fabrication. Temperature conditioning was implemented for all specimens before mechanical testing, with a holding time of no less than 4 h. The standard static test was carried out at 15 °C, and supplementary static strength characterizations were performed at four additional temperatures: −10, 0, 30, and 45 °C. Owing to the mismatch in thermal conductivity between the concrete base and SMA-13 overlay, direct measurement of internal specimen temperature is impractical. A dummy composite beam of identical dimensions (300 mm × 100 mm × 100 mm), instrumented with temperature sensors, was therefore placed in the environmental chamber (Shanghai Yiheng Scientific Instruments Co., Ltd., Shanghai, China) together with the test specimens to monitor core temperature stabilization. Two calibrated Pt100 RTDs (±0.1 °C accuracy, Omega Engineering Inc., Norwalk, CT, USA) were fixed to the dummy beam: one on the top surface, and the other at the geometric midpoint, which aligns with the interlayer interface 50 mm below the surface. Pre-test calibration showed that after 4 h of conditioning, the temperature deviation between the core and surface of the dummy beam relative to the target setpoint was less than ±0.5 °C. A further 30 min holding period was required once this equilibrium was reached, after which mechanical testing could begin. All tests were performed on an MTS fatigue testing machine (MTS Systems Corporation, Eden Prairie, MN, USA). Each beam specimen was carefully positioned on the two roller supports of the three-point bending fixture, with a support span (L) of 250 mm. A laser alignment device (Robert Bosch Tool Corporation, Mount Prospect, IL, USA) was used to calibrate the specimen position, ensuring that the centerline of the pre-cut notch on the bottom C40 concrete side coincided with the central axis of the loading indenter. This setup guaranteed consistent stress concentration at the simulated reflective crack location. The test was conducted under displacement-controlled loading at a rate of 1 mm/min (at 15 °C). Load (F) and mid-span deflection (D) data were synchronously recorded by the test system. Loading was terminated when the instantaneous load dropped below 10% of the peak load, which was defined as the macroscopic failure state of the specimen. The nominal flexural tensile fracture strength R of pre-notched layered composite beams was calculated via Equation (1):

(1) $$R=\frac{3LF}{2bH^{2}}$$

where F is the peak load at specimen failure (N); H is the actual height of the specimen at the mid-span cross-section (mm); L is the span length of the specimen (250 mm); and b is the width of the specimen at the mid-span cross-section (mm). Four parallel specimens were fabricated and tested under each test condition. Grubbs’ test at a significance level of α = 0.05 was applied to identify outliers in the peak load dataset. Given the small sample size of four specimens per group, the corresponding critical Z-value is very conservative, and as a result, no data points were removed from the final analysis. The arithmetic mean value for each test group was computed and used as the reference baseline for cross-group comparison.

#### 2.3.2. Cyclic Three-Point Bending Fatigue Test

**Figure 1:**
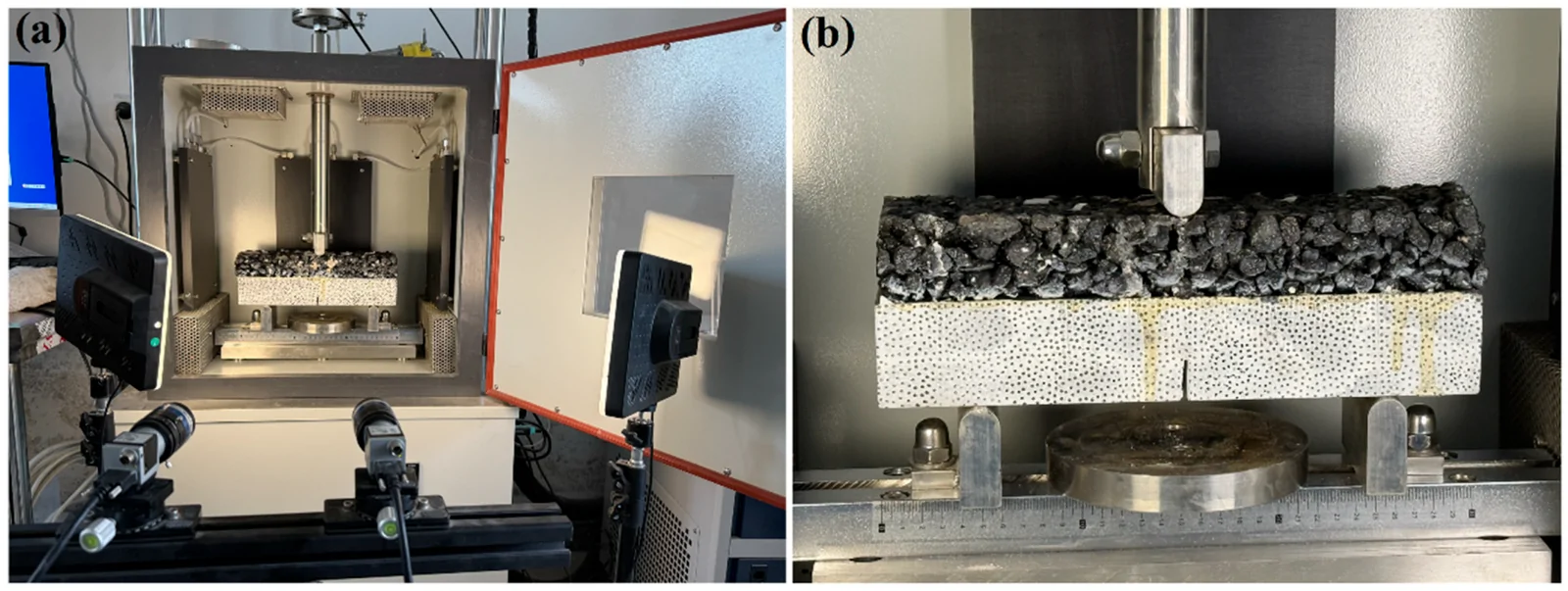
Three-point bending test setup for rigid-flexible composite beams: (**a**) MTS loading device; (**b**) composite beam specimen and magnified view of the speckle pattern.

The formation of reflective cracks in asphalt pavements is essentially a process of cumulative fatigue damage induced by long-term cyclic dynamic loading. In this study, laboratory accelerated fatigue tests were performed to investigate the variation in fatigue life (N_f_) of specimens with different crack-resistant interlayer configurations. All fatigue tests were carried out on an MTS fatigue testing machine under stress-controlled loading mode, as shown in Figure 1. To closely replicate the pulsed loading characteristics of vehicle loads in tunnel environments, a half-sine (haversine) waveform was adopted. The stress ratio R (ratio of minimum load to maximum load) was set to 0.1 to avoid secondary impact caused by detachment of the loading indenter from the specimen surface during the high-speed unloading phase. To systematically evaluate the combined effects of temperature, loading frequency, and stress level on the fatigue life of the interlayer interface, three key test parameters were varied: temperature, stress level and loading frequency. Four replicate specimens were tested for each condition. After removing outliers, the arithmetic mean was taken as the representative result for each group. Outliers were removed using Grubbs’ test at a significance level of α = 0.05. The coefficient of variation (CV) for all valid samples was maintained below 15%. Error bars in all figures represent ±1 standard deviation.

(1) Test temperature

Five test temperatures were set: −10 °C, 0 °C, 15 °C, 30 °C and 45 °C. This range covers the typical interlayer temperature range experienced by asphalt pavements in most regions of China during service. For all cyclic fatigue tests conducted at the five temperatures (−10, 0, 15, 30, and 45 °C), the same 4-h isothermal conditioning and dummy-beam temperature verification method was used, consistent with the static test procedure in Section 2.3.1. Given that fast temperature changes produce significant thermal lag across the specimen cross-section, cyclic loading was not initiated until both the surface and embedded interlayer sensors on the dummy beam stabilized within ±0.5 °C of the chamber temperature. The chamber remained closed and temperature-controlled without interruption for the entire duration of each fatigue test. Li et al. [22] conducted thermal fatigue cracking tests on asphalt composite structures over a wide temperature range with cyclic temperature fluctuations. They analyzed the evolution of internal stress relaxation and accumulation within the temperature range of −20 °C to 0 °C, and confirmed that extreme temperature environments accelerate fatigue damage. Chen et al. [23] combined high-frequency fatigue loading tests with numerical simulations to examine the spatial distribution of stress at different temperatures, characterized crack propagation patterns during temperature variation, and discussed the influence of thermal loading on the damage severity of asphalt pavements. Their results showed that the surface course is most affected by temperature changes, while the base course is least affected. Higher ambient temperature leads to wider crack propagation within the pavement structure and a faster propagation rate, accelerating the fatigue damage rate of the pavement structure.

(2) Stress level

The stress level (S) is defined as the ratio of the peak cyclic load applied in fatigue tests (*F*_applied_) to the static ultimate fracture load measured at the same temperature (*F*_static_), as shown in Equation (2):

(2) $$S=\frac{F_{applied}}{F_{static}}$$

To ensure accurate stress level determination, static three-point bending tests were conducted at each test temperature (−10, 0, 15, 30, and 45 °C) prior to fatigue testing. Tests were performed under displacement control at a loading rate of 1 mm/min, as specified in Section 2.3.1. The resulting temperature-dependent static peak loads served directly as the reference for normalising stress levels in fatigue tests at the corresponding temperatures. Wang et al. [24] performed indirect tensile fatigue tests on asphalt mixtures under constant temperature (20 °C) and constant frequency (10 Hz) conditions, with stress levels set to 0.3, 0.4, 0.5 and 0.6. The test results indicated that the evolution of vertical displacement and fatigue degradation of the material within this stress range can effectively reflect the nonlinear damage behavior under the alternating tensile-compressive stress field in actual pavement. In addition, mesoscale shear failure is the dominant factor controlling the early failure of specimens at high stress levels. Accordingly, three stress levels (0.3, 0.4 and 0.5) were adopted in this beam fatigue test to evaluate the fatigue performance of specimens under different loading conditions. This range covers the interlayer shear stress conditions of actual pavement structures under various traffic load levels.

(3) Loading Frequency

Vehicle speed directly determines the loading duration applied to the pavement structure and the interval between adjacent load pulses: higher vehicle speed corresponds to shorter single-load duration and longer loading intervals. Four loading frequencies were adopted in this test: 1 Hz, 5 Hz, 10 Hz and 15 Hz [25,26,27]. According to existing domestic and international studies, a loading frequency of 1 Hz corresponds to a driving speed of approximately 20 km/h, 5 Hz to 40 km/h, 10 Hz to 60 km/h, and 15 Hz to 80 km/h. This frequency range covers the typical operating speed range of expressways in China.

A dual failure criterion was adopted to determine the fatigue life (N_f_) of each specimen. Failure was deemed to occur when either of two conditions was met. First, the composite specimen developed a visible macroscopic through-thickness crack and suffered complete structural fracture. Second, the dynamic effective stiffness (E) of the specimen degraded to 50% of its initial stiffness (E_0_). Effective stiffness per loading cycle was computed as the ratio of the peak cyclic load to the corresponding peak mid-span deflection, given by E = F_max_/D_max_. Initial stiffness E_0_ was taken as the stiffness at the 100th loading cycle, to eliminate artifacts caused by initial specimen seating and compaction. This dual-criterion approach is mechanically essential for comparing material systems with markedly different constitutive responses. Experimental observations confirmed that semi-brittle, high-strength EEA specimens failed solely according to the first criterion. Sudden macroscopic splitting occurred in these specimens due to localized stress concentration, before stiffness degradation reached the 50% threshold. For highly ductile rubberized asphalt specimens, by contrast, failure was governed by the second criterion, following extensive cohesive energy dissipation and without abrupt structural fracture. Both criteria mark the functional end-of-life of the interlayer, when load-transfer capacity is lost, and thus ensure direct comparability of the decomposed life metrics (N_c_) and (N_a_) across different failure modes. To eliminate spurious modulus degradation caused by test apparatus running-in and specimen compaction in the early loading stage, the stiffness modulus at the 100th loading cycle was taken as the initial value. Recent studies on composite interface fatigue have shown that adopting a dual failure criterion has practical engineering value for distinguishing the mechanical differences between rigid and ductile materials [28]. Abualia et al. [29] verified through comparative rheological and fatigue tests that high-toughness materials with strong viscoelastic deformation capacity (e.g., rubberized asphalt) mainly undergo long-term cohesive energy dissipation during damage evolution, and can complete the full fatigue process steadily. Such materials usually trigger the 50% modulus decay criterion first without instantaneous fracture. In contrast, semi-brittle high-strength interfacial materials (e.g., pure epoxy systems) are susceptible to sudden macroscopic splitting failure before the modulus drops to 50%, due to localized stress concentration. The dual failure criterion compensates for the limitation of a single evaluation index when applied to interfaces with different ductile-brittle properties, and ensures the rigor and reliability of fatigue life boundary definition for composite specimens.

Given the large volume of data generated in high-frequency fatigue tests, a tailored data acquisition scheme was adopted to calculate dissipated energy while avoiding redundant sampling. For the first 100 loading cycles, data were recorded on a cycle-by-cycle basis to capture the initial compaction and stress redistribution processes. During the steady-state damage evolution phase, complete load–deflection hysteresis loops were acquired at logarithmically spaced intervals (e.g., 200, 500, 1000 cycles). The energy dissipated per cycle (*w*_i_) was computed as the area enclosed by the corresponding hysteresis loop. Since damage evolution remains highly linear and stable in this phase, the trapezoidal rule was used to interpolate energy values for unsampled cycles, introducing negligible error. The cumulative dissipated energy (*W*_c_) of the test specimen is calculated by discrete numerical integration, as given in Equation (3):

(3) $$W_{c}=\sum \frac{1}{2}\left(w_{i}+w_{i+1}\right)\left(N_{i+1}-N_{i}\right)$$

To eliminate interpolation errors during the highly nonlinear final failure phase, the control system was configured to automatically switch back to continuous, cycle-by-cycle sampling once the normalized stiffness ratio (E/E_0_) dropped below 0.6. The total cumulative dissipated energy (*W*_c_) was then obtained by discrete numerical integration of *w*_i_ over the full fatigue life (N_f_). To isolate the effect of total cycle count from the intrinsic damping capacity of the material, the average dissipated energy per cycle (*w*_avg_), defined as w_avg_ = *W*_c_/*N*_f_, was adopted as the key normalized parameter for evaluating interfacial fracture toughness. Li et al. [30] pointed out in their study on the residual fatigue life of asphalt mixtures that calculating cumulative fatigue damage based on dissipated energy extracted from logarithmically spaced hysteresis loops can effectively improve data processing efficiency, and fully reproduce the nonlinear degradation process of mixtures from the viscoelastic response stage to the microcrack propagation stage.

#### 2.3.3. Optical Crack Monitoring and Stage Decomposition Criteria

Accurate cycle-resolved crack tip tracking is a prerequisite for the two-stage fatigue life decomposition into N_c_ and N_a_. For this purpose, a high-resolution optical monitoring system was established. The side surface of each composite beam was sprayed with matte white paint and engraved with a 5 mm × 5 mm high-contrast reference grid. A sharp horizontal reference line was marked exactly at the concrete–asphalt bimaterial interface at a depth of 50 mm from the bottom of the beam.

The monitoring system consisted of a high-speed industrial CMOS camera (4096 × 2160 pixels, Basler AG, Ahrensburg, Germany) equipped with a macro lens(Nikon Corporation, Tokyo, Japan), mounted orthogonally to the specimen observation face. The system provided a 100 mm × 100 mm field of view, corresponding to a spatial resolution of 0.025 mm/pixel. An external trigger linked to the MTS load cell was used to synchronize image capture. Frames were acquired precisely at the tensile peak of each haversine loading cycle to maximize crack opening visibility. Continuous image recording was applied for the first 100 cycles, after which images were captured at discrete logarithmic intervals matching the sampling schedule of hysteresis loop data, such as 200, 500 and 1000 cycles.

The crack tip was identified numerically as the highest coordinate of the physical fracture discontinuity with a horizontal opening displacement greater than 0.05 mm, or 2 pixels. The base course crack propagation life N_c_ was defined as the loading cycle at which the optically tracked crack tip intersected the 50 mm interfacial reference line. When interface penetration fell between two adjacent logarithmic sampling points, N_c_ was determined via linear interpolation using the crack propagation rate calculated from neighbouring frames. The interface-overlay propagation life N_a_ was then computed as the difference between the total fatigue failure life N_f_ and N_c_.

## 3. Results and Discussion

### 3.1. Evaluation of Optimum Application Rate and Static Ultimate Flexural Tensile Capacity of Interlayer Coatings

**Figure 2:**
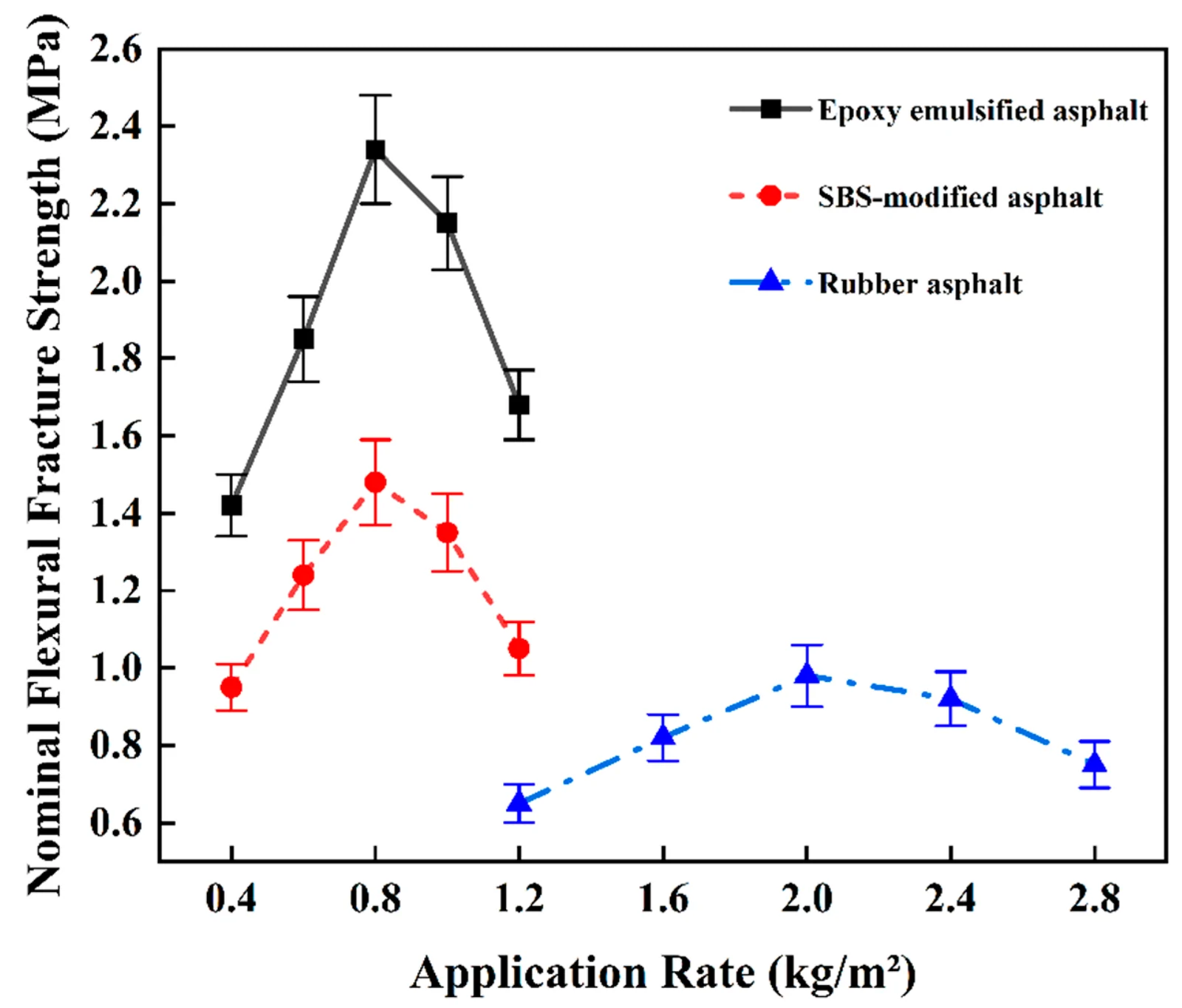
Effect of application rate on interfacial shear strength of different interfacial coatings.

During the service life of tunnel composite pavements, heavy wheel loads passing over rigid base joints or microcracked regions subject the interlayer interface to a coupled bending-shear stress state combining Mode I (tensile opening) and Mode II (in-plane shear) [31]. To characterize the interlayer shear performance under this coupled stress state, static three-point bending tests were conducted on rigid-flexible composite beams with pre-cut bottom notches, to quantify the interfacial shear strength of three coating materials at varying application rates. As shown in Figure 2, the shear strength of epoxy emulsified asphalt (EEA), SBS-modified asphalt and rubberized asphalt all follows an inverted U-shaped trend, increasing first and then decreasing with rising application rate. This confirms that all three interfacial bonding layers have an optimal application rate corresponding to their peak shear strength. Specifically, both EEA and SBS-modified asphalt reached their peak strength at an application rate of 0.8 kg/m^2^, with values of 2.35 MPa and 1.48 MPa, respectively. Rubber-modified asphalt produced the lowest static nominal flexural tensile fracture strength of 0.98 MPa. The optimum application rate corresponding to this peak strength was 2.0 kg/m^2^, notably higher than the 0.8 kg/m^2^ determined for the other two coating materials. The interfacial bonding performance of all three anti-cracking materials is strongly governed by the application rate. At low application rates, EEA, SBS-modified asphalt and rubberized asphalt cannot fully wet the roughened concrete surface, leading to discontinuous coating films and a reduced effective interfacial bonding area. Under three-point bending loading, stress concentration develops at crack tips, which readily triggers premature interfacial delamination failure. At the optimal application rate, the coating forms a continuous and uniform adhesive film, which gives full play to the mechanical interlocking and adhesive effects at the interface, resulting in the peak macroscopic shear strength [32]. Once the application rate exceeds the optimal threshold, excess asphalt accumulates at the interface, forming a thick free asphalt film. During shear deformation, the lubricating effect of this film weakens the interlayer slip resistance, and the interfacial failure mode transitions from adhesive failure at the concrete–coating interface to cohesive failure within the asphalt film, ultimately reducing the macroscopic shear strength [33].

The differences in peak shear strength and optimal application rate among the three materials are fundamentally determined by their chemical composition and mesostructural characteristics. The superior shear resistance of EEA stems from the dense three-dimensional cross-linked thermoset network formed by the waterborne epoxy resin (E-51) upon curing. This network not only enhances the cohesive modulus of the material itself, but also forms stable chemical bonds with the cement concrete substrate [34]. For the rubberized asphalt system, the high content of swollen crumb rubber particles leads to a relatively high zero-shear viscosity. During spraying and compaction, a greater amount of base asphalt is required to encapsulate the rubber particles and fill the rough texture of the concrete surface. This volume-filling effect induced by particle swelling is the main physical mechanism underlying the significantly higher optimal application rate of rubberized asphalt compared with EEA and SBS-modified asphalt [35].

### 3.2. Static Flexural Fracture Behavior of Interfacial Coatings

**Figure 3:**
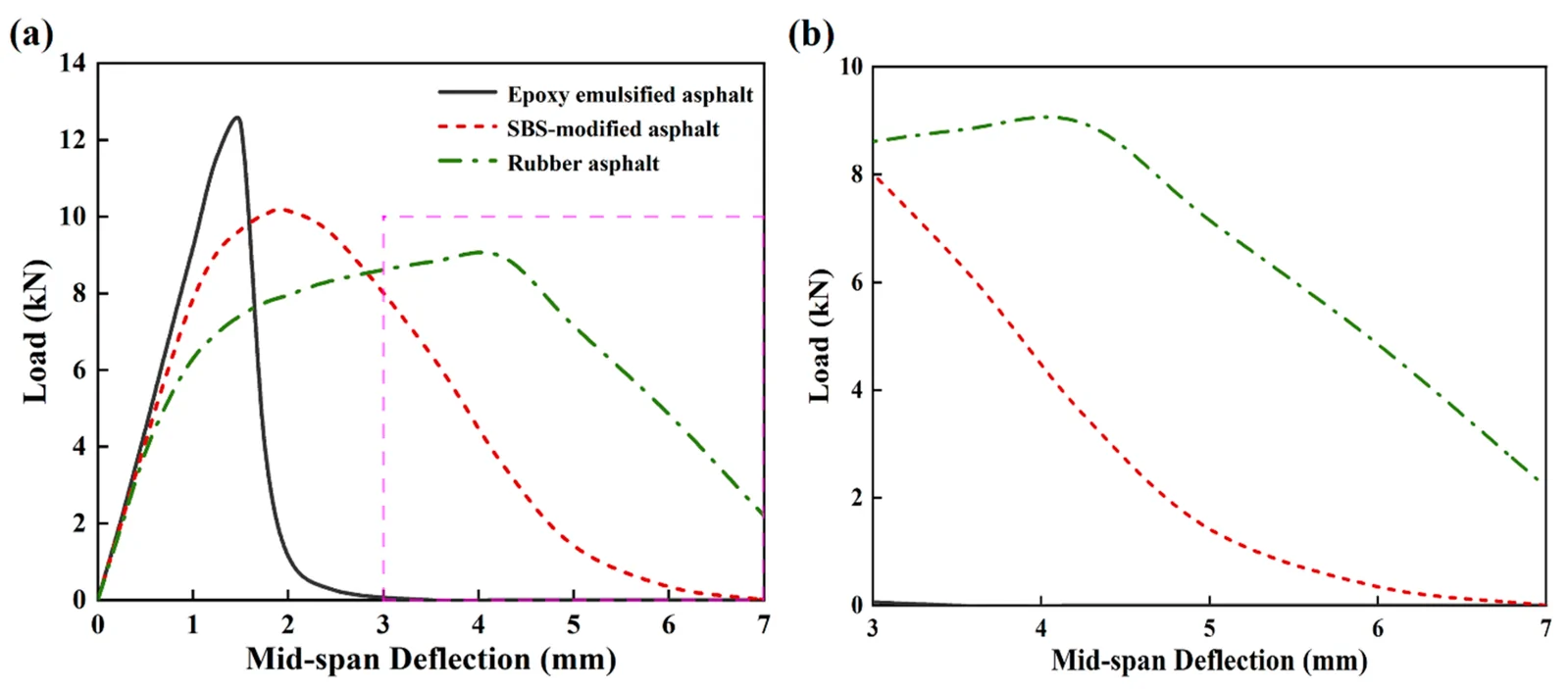
Static three-point bending load–displacement (P–D) curves and local magnifications for composite beams with three interfacial coatings: (**a**) full load–displacement response; (**b**) magnified view of the post-peak segment.

To investigate the crack resistance and energy dissipation behavior of different interfacial coating materials, comparative analysis was performed based on static three-point bending fracture test results. Figure 3 presents the full load–midspan deflection (P–D) curves and magnified post-peak segments for composite beams with a 15 mm deep pre-cut notch, prepared with EEA, SBS-modified asphalt and rubberized asphalt respectively. As shown in Figure 3a, the initial bending stiffness (slope of the P–D curve before the peak) of the three groups of specimens was largely consistent during the initial linear elastic ascending phase. This consistency arises because, prior to crack propagation reaching the interfacial layer, the overall bending stiffness of the composite structure is dominated by the 50 mm thick C40 cement concrete base course. The thin flexible bonding layer, only a few millimeters thick, makes a negligible contribution to global stiffness.

As the load approached its peak and entered the post-peak softening phase, differences in the constitutive properties of the interfacial coatings began to exert a pronounced influence on structural failure modes. EEA specimens achieved the highest peak load-bearing capacity, with a maximum load (F_max_) of approximately 12.6 kN, but load-bearing capacity declined sharply once the peak was exceeded. As illustrated in Figure 3b, load-bearing capacity was almost fully exhausted at a midspan deflection below 2.5 mm, which is characteristic of high-strength, low-ductility brittle fracture. This behavior stems from the high-modulus cross-linked network formed after EEA curing: while this structure delivers strong interlayer bond strength, it offers limited plastic deformation capacity. When crack tip stress from the base course is transferred to the interface, EEA cannot effectively dissipate stress through deformation, allowing cracks to propagate rapidly upward into the SMA-13 surface course.

By contrast, rubberized asphalt composite beams showed the lowest peak load (approximately 9.1 kN) but featured an extended post-peak softening plateau on the P–D curve (Figure 3b). At a midspan deflection of 7 mm, these specimens still maintained a residual load-bearing capacity above 2 kN, reflecting a clear low-stiffness, high-ductility mechanical response. This superior energy dissipation capacity is primarily attributed to the high content of swollen crumb rubber particles in the rubberized asphalt. When cracks from the base course propagate upward to the anti-cracking interfacial layer, the rubber particles undergo shear deformation thanks to their excellent elastic buffering performance. This process generates microporous structures within the material, dissipating crack propagation energy in the form of heat and deformation work. As a result, crack tip stress concentration is alleviated, crack growth rate is reduced, and upward propagation of reflective cracks is effectively suppressed. SBS-modified asphalt specimens exhibited intermediate load-bearing capacity and ductility, with an elastoplastic failure mode and moderate energy dissipation performance.

**Figure 4:**
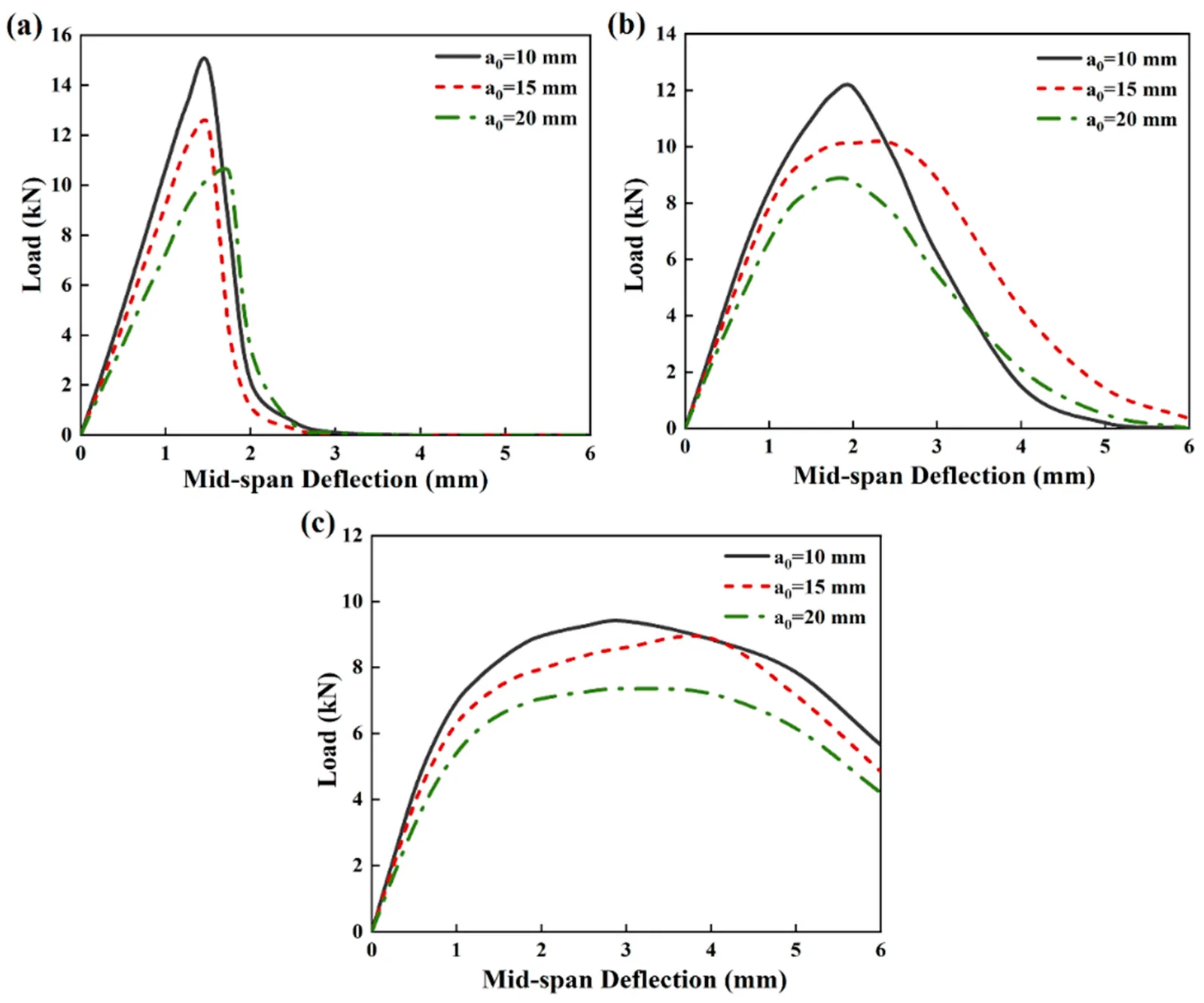
Three-point bending load–displacement curves of composite beams with different initial notch depths a_0_: (**a**) epoxy emulsified asphalt; (**b**) SBS-modified asphalt; (**c**) rubberized asphalt.

The initial notch depth (a_0_) in the concrete substrate directly dictates the magnitude of the initial crack-tip stress intensity factor, which in turn governs the apparent fracture resistance of the composite system. To quantitatively assess the structural stability of composite beams under varying levels of pre-existing damage, peak fracture loads corresponding to a_0_ = 10, 15, and 20 mm were compared across all test groups, as shown in Figure 4. An increase in a_0_ reduces the effective uncracked ligament area, leading to a general decline in peak loads for all specimen groups. However, quantitative sensitivity analysis reveals distinct material-dependent responses. When the notch depth increases from 10 mm to 20 mm, the rigid EEA composite beam undergoes a sharp 32% reduction in peak load, reflecting its high sensitivity to initial flaw size. By comparison, the rubberized asphalt composite beam exhibits a markedly smaller peak load decrease of approximately 25% under the same notch increment, retaining a residual peak load of around 7.4 kN and featuring an extended post-peak softening plateau. This load retention capacity numerically confirms that the high ductility of rubberized asphalt blunts crack-tip stress singularities, and accordingly lowers the sensitivity of the composite structure to the size of base-course defects.

### 3.3. Variation Characteristics of Interlayer Fatigue Life Under Varying Temperature and Loading Conditions

#### 3.3.1. Coupling Effects of Temperature and Loading Frequency

**Figure 5:**
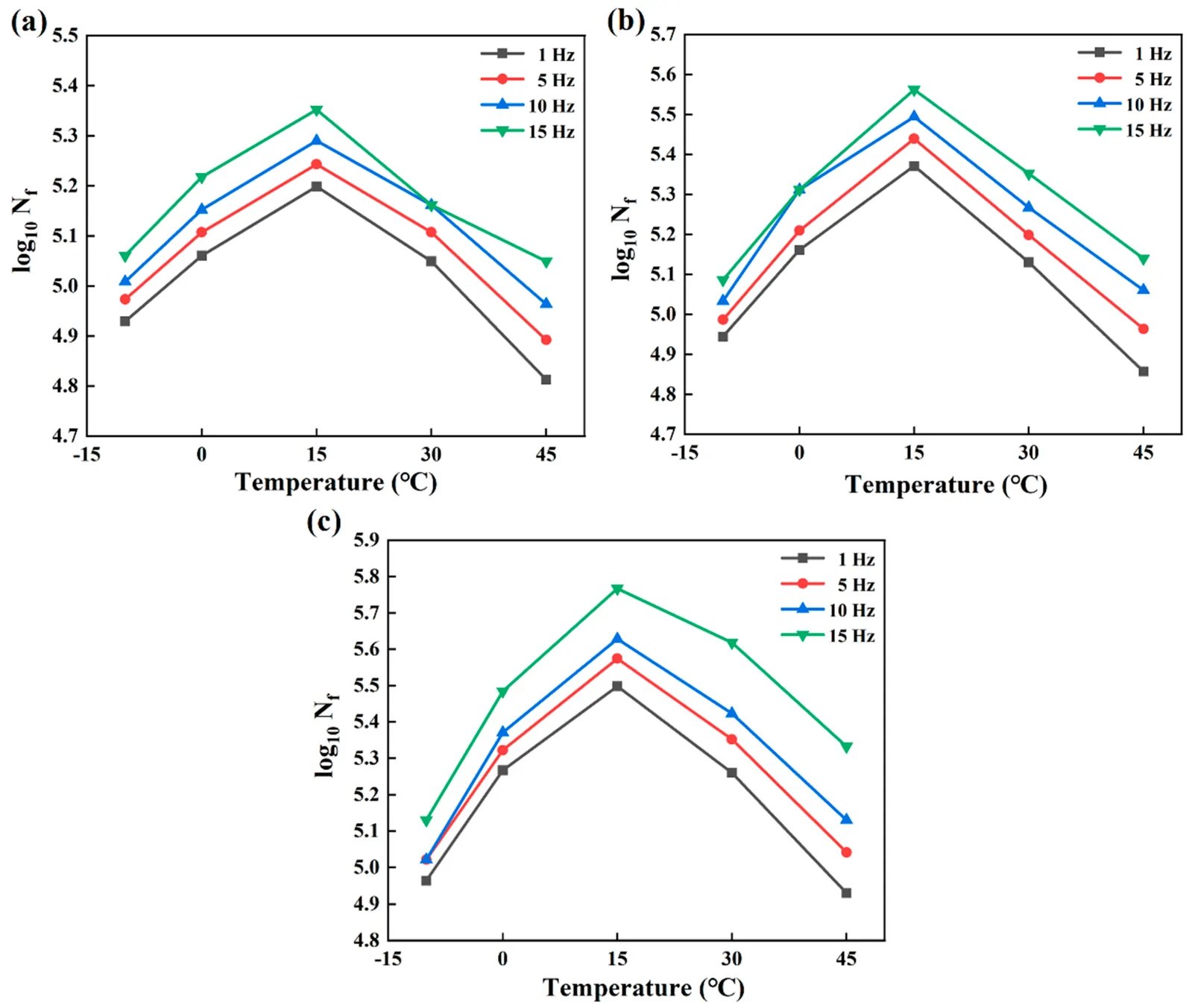
Fatigue life as a function of temperature for three interfacial coatings under different loading frequencies: (**a**) epoxy emulsified asphalt; (**b**) SBS-modified asphalt; (**c**) rubberized asphalt.

Figure 5 presents the variation curves of the logarithmic fatigue life (log N_f_) for three types of interfacial anti-cracking coatings under different temperature and loading frequency conditions. When a fixed stress amplitude was applied in the test, the fatigue life of all three materials did not decrease monotonically with increasing temperature. Instead, all samples followed an inverted V-shaped pattern, with the maximum fatigue life occurring at room temperature (15 °C). In the low-temperature range of −10 °C to 0 °C, the asphalt binder remains in a typical glassy state and transitions gradually toward a viscoelastic state. At −10 °C, the extreme low temperature end, the material exhibits very high bulk stiffness and nearly negligible stress relaxation capacity. When the temperature rises to 0 °C, the material develops limited viscoelastic characteristics, while the interlayer coating stiffness remains comparatively high. In this temperature interval, the superposition of thermal and mechanical stresses readily induces stress concentration at the crack tip, yet the material’s viscous flowability is insufficient to fully dissipate the resulting destructive energy. This mismatch between deformation recovery capacity and stress concentration prevents effective suppression of microcrack initiation and brittle propagation, leading to overall low fatigue durability across all specimens in the low-temperature range. As temperature increases to 15 °C, the fatigue life of all three materials follows an inverted V-shaped trend, reflecting a temperature-dependent competitive mechanism between material strength, brittle fracture, and viscous flow failure. At low temperatures, the material is prone to brittleness, and stress concentration readily triggers brittle propagation of microcracks. At elevated temperatures, the asphalt matrix shifts to a viscous flow state, and irreversible plastic deformation degrades fracture resistance. At 15 °C, the material’s macroscopic flexural tensile stiffness and viscoelastic energy dissipation capacity reach a favorable balance, yielding peak fatigue life. In the high-temperature range of 30 °C to 45 °C, interlayer materials with different chemical systems exhibit distinctly different mechanical degradation mechanisms. As thermoplastic materials, SBS-modified asphalt and rubber-modified asphalt undergo a gradual transition of the asphalt matrix to a viscous flow state at high temperatures. Extensive chain slippage occurs within the internal polymer network, and irreversible plastic strain accumulates with each loading cycle, causing rapid deterioration of fracture resistance. In contrast, epoxy-emulsified asphalt (EEA) forms a three-dimensional crosslinked thermoset network after curing, and no physical phase transition to a viscous flow state occurs over this temperature range. The decline in EEA fatigue life at high temperatures is primarily attributed to thermally induced free volume expansion of the crosslinked network and relaxation of polymer segments, which significantly reduce the material’s macroscopic mechanical modulus and interlayer mechanical interlocking. Under high-frequency stress, this modulus reduction ultimately triggers interlayer debonding and localized brittle fracture.

As the loading frequency increased from 1 Hz to 15 Hz, the fatigue life of the specimens improved significantly. This phenomenon is primarily associated with the viscoelastic constitutive behavior of asphalt composite materials. The higher the loading frequency, the shorter the duration of each half-sine loading pulse; this effectively restricts viscous flow deformation and irreversible plastic strain accumulation within the interlayer coating. Post-loading deformation recovery is dominated by elastic rebound, which slows the progression of interfacial slippage and damage at the interlayer interface. The test results show that the fatigue life of the three interfacial materials—EEA, SBS-modified asphalt, and rubberized asphalt—increased by 1.8 to 2.5 times overall.

#### 3.3.2. Effect of Stress Level

**Figure 6:**
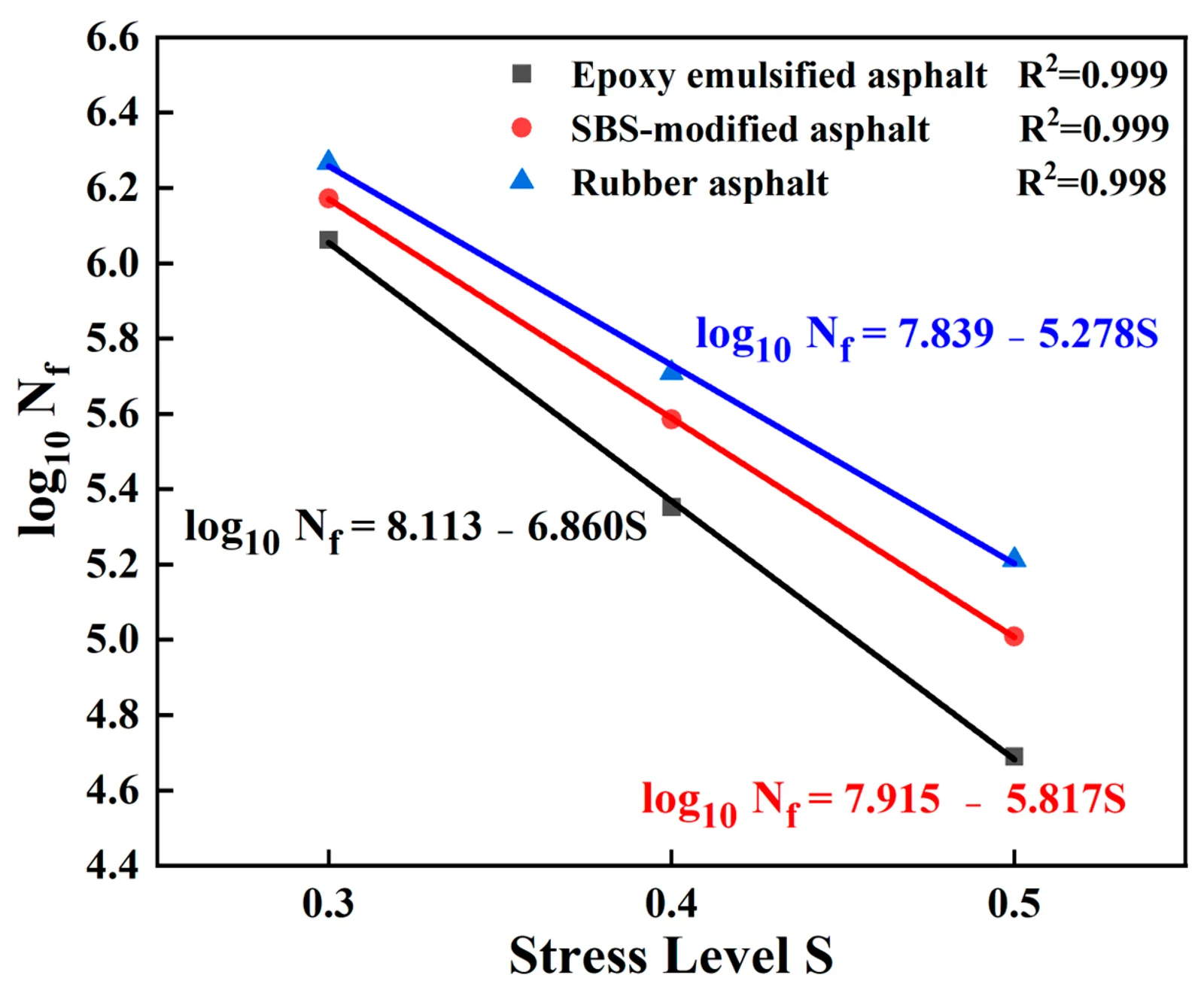
Fatigue life as a function of stress level for the three interfacial coatings.

Traffic overload, corresponding to an increase in stress level S, is a major external factor accelerating fatigue failure of interfacial coatings. Fatigue life data at the reference temperature were extracted to establish a fatigue life prediction equation in a log-log coordinate system. As shown in Figure 6, logarithmic fatigue life (log_10_N_f_) shows a good linear negative correlation with stress level S, with all coefficients of determination (R^2^) above 0.998. This trend agrees well with the classical S-N fatigue law. Comparison of the fitted line slopes indicates that EEA has the largest absolute stress sensitivity coefficient (−6.860), followed by SBS-modified asphalt (−5.817). The curve for rubberized asphalt is the flattest, with a sensitivity coefficient of −5.278. These differences suggest that the high-stiffness, relatively brittle EEA generates a substantially increased crack tip driving force under high stress amplitudes, leading to much higher sensitivity to overload conditions. In contrast, the abundant crumb rubber particles from recycled tires in rubberized asphalt form a flexible high-elastic network, which effectively dissipates crack tip stress and reduces the material’s sensitivity to extreme loads. This gives rubberized asphalt better structural adaptability under heavy-load and high-stress service conditions.

#### 3.3.3. Normalized Stiffness Degradation and Hysteretic Energy Dissipation Characteristics

**Figure 7:**
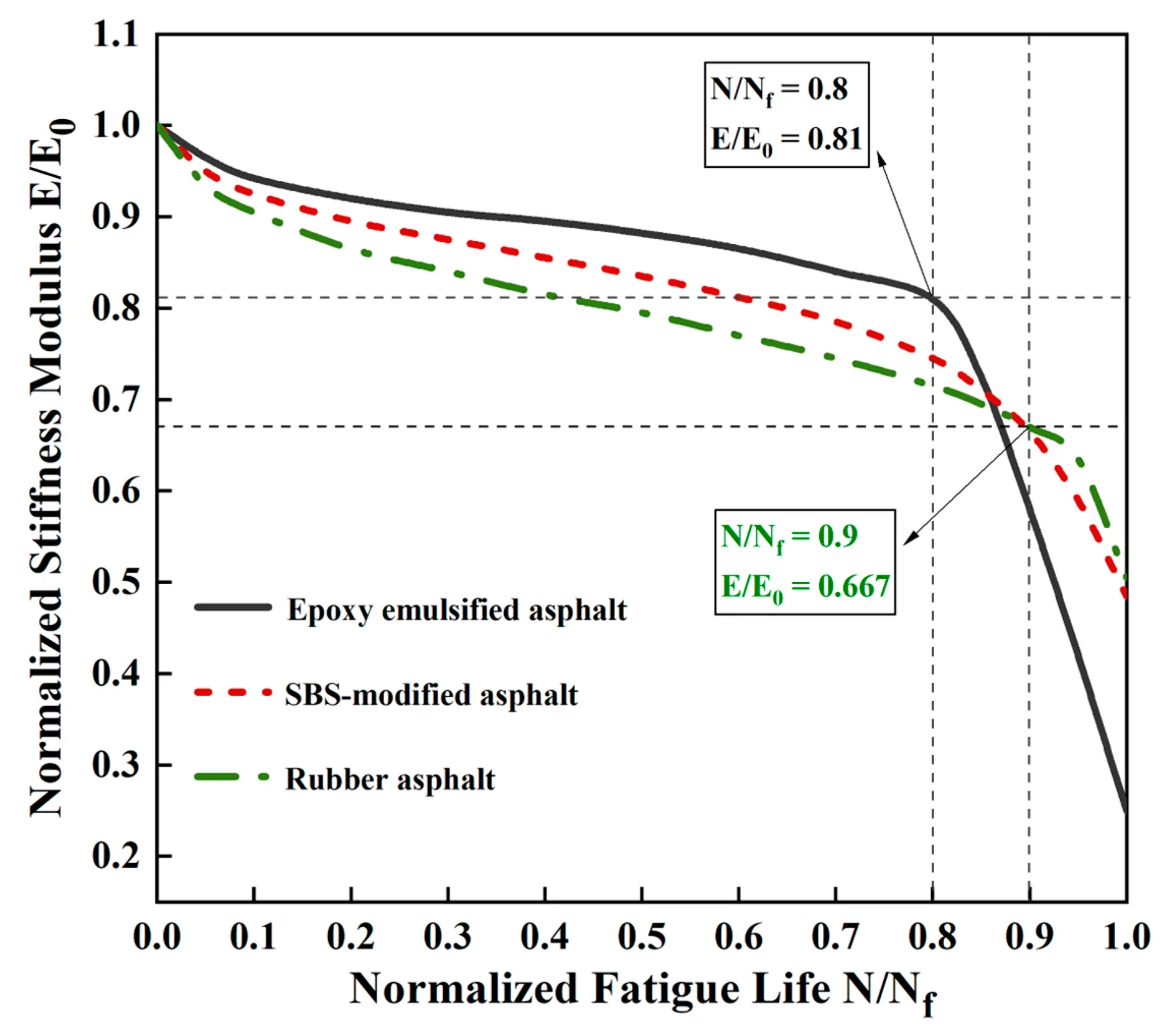
Normalized stiffness degradation curves for the three interfacial coatings.

Stiffness degradation is a direct mechanical indicator reflecting fatigue damage accumulation in tunnel composite pavement structures. To eliminate interference from differences in the initial stiffness (E_0_) of the three interfacial coatings on damage evolution patterns, normalized stiffness ratio (E/E_0_) curves were plotted against normalized fatigue life (N/N_f_). As shown in Figure 7, under alternating bending-shear stress, all three interfacial anti-cracking coatings follow a typical three-stage nonlinear degradation pattern: initial compaction and microcrack initiation, stable damage propagation, and accelerated failure. The three coatings differ notably in the position of inflection points and degradation rates due to their inherent mechanical properties. EEA shows high-strength brittle fracture behavior: its stiffness decreases slowly in the first 80% of fatigue life cycles, with residual stiffness remaining at 81% of the initial value. This behavior arises from the high-modulus cross-linked network formed by cured epoxy resin, which effectively suppresses microcrack opening and interfacial slip in the early fatigue stage. Once the damage threshold is exceeded, however, microcracks propagate rapidly and coalesce within the rigid matrix, causing a sharp drop in the stiffness ratio. This indicates that brittle materials such as EEA cannot effectively dissipate crack tip fracture energy through plastic deformation in the late stage of crack propagation.

By contrast, rubberized asphalt has excellent viscoelastic toughness and a high deformation limit, leading to a longer stable damage stage with a more gradual degradation rate. At a normalized life N/N_f_ = 0.9, its normalized stiffness ratio remains at 0.667. Even after entering the final failure stage, the stiffness decline rate is still markedly slower than that of EEA. This performance is attributed to the fully swollen rubber particles in rubberized asphalt, which form a flexible buffer structure. Under repeated bending-shear loads, these particles convert external load work into heat dissipation through viscoelastic deformation and intermolecular friction, thereby alleviating crack tip stress concentration, retarding crack growth, and preventing sudden structural failure. SBS-modified asphalt exhibits intermediate degradation behavior and damage tolerance, with a conventional elastoplastic fatigue response.

**Figure 8:**
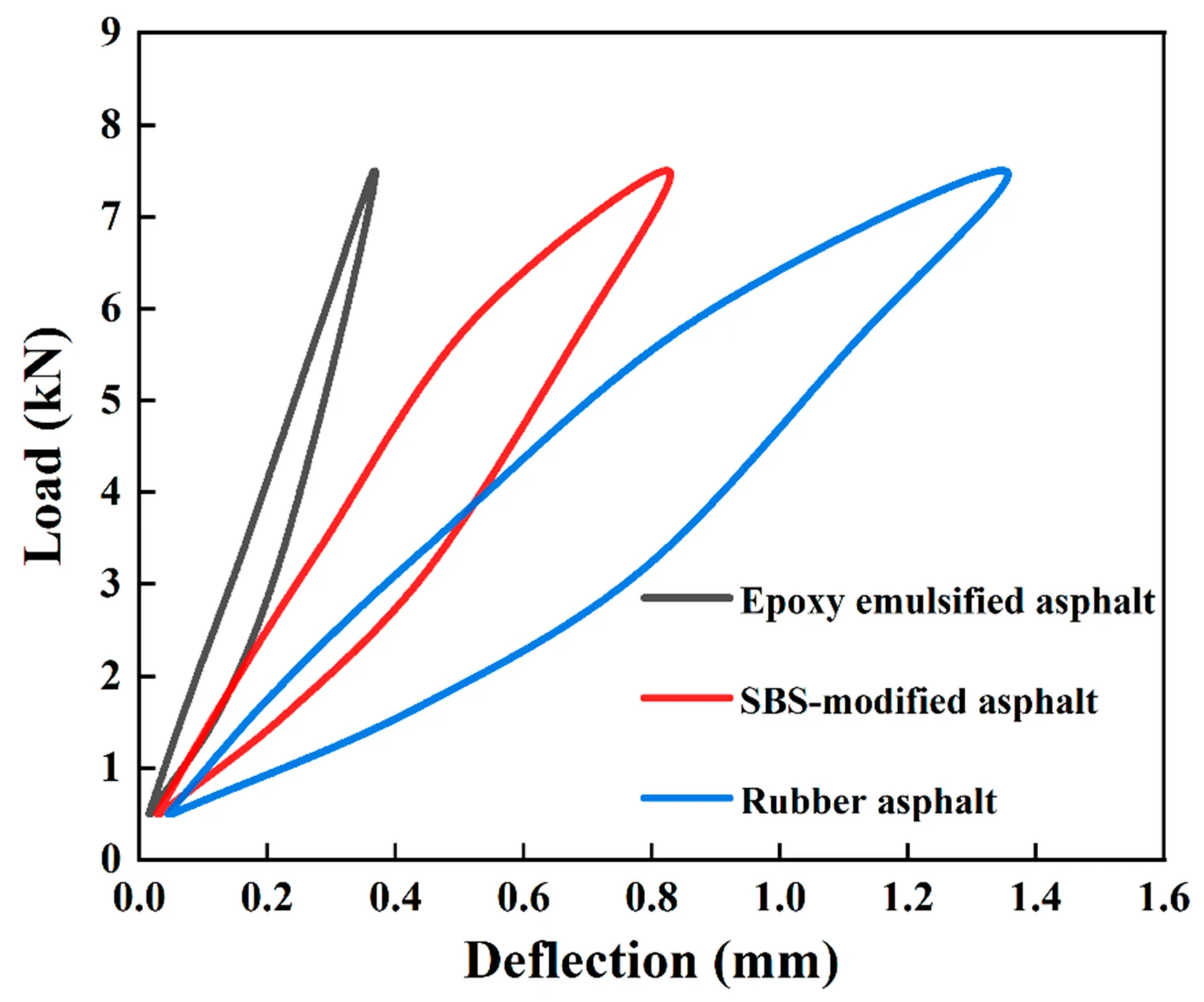
Load–deflection (P–D) hysteresis loops of composite beam specimens with three interfacial coatings.

To characterize the dynamic mechanical behavior of the three interfacial anti-cracking coatings under coupled bending-shear stress, load–deflection (P–D) hysteresis loops of the composite beams were extracted from test data corresponding to the stable damage propagation stage of cyclic fatigue tests. As shown in Figure 8, the three interfacial coatings differ notably in hysteresis loop morphology, which reflects their distinct constitutive properties. The EEA sample features a steep tangent stiffness and a small enclosed loop area. This indicates that under cyclic bending-shear loading, deformation of the material is dominated by elastic reversible deformation with weak viscous energy dissipation, showing typical high-strength brittle characteristics. When the load acts through the base course notch, EEA cannot buffer the instantaneous impact via plastic deformation. Most of the input mechanical energy is converted into crack tip fracture driving force, which readily induces localized stress concentration. This observation aligns with the results reported by Yang et al. [36], who noted that rigid epoxy interfaces have limited energy dissipation capacity, and undissipated strain energy accelerates crack propagation, eventually triggering abrupt stiffness degradation.

By comparison, the hysteresis loop of the rubberized asphalt composite exhibits a markedly lower tangent stiffness and a larger enclosed area, characteristic of a pronounced viscoelastoplastic mechanical response. To quantify this energy dissipation performance independent of prolonged fatigue life, the average dissipated energy per cycle (w_avg_) was computed. The rubberized asphalt composite delivers an average energy dissipation of 0.375 J per cycle, a value markedly higher than the 0.120 J/cycle measured for the brittle EEA system. Previous studies on the crack mitigation mechanism of high-toughness modified asphalt, such as those by Mahmoud [37] and Li [38], have also shown that this damping effect from large-deformation hysteretic energy dissipation can macroscopically reduce the driving force of crack propagation into the surface course and delay crack penetration. A mechanistic hypothesis is proposed that this macroscopic energy dissipation arises from the internal swelling and viscoelastic flow of embedded crumb rubber particles. Under cyclic shear deformation, these elastomeric domains generate localized internal friction, which converts crack-tip mechanical strain energy into dissipated heat. While direct validation of the underlying internal cavitation and micropore formation mechanisms will require further non-destructive micro-imaging investigations, the macroscopic hysteresis results clearly demonstrate that elevated per-cycle energy dissipation consistently mitigates localized stress concentration, and is directly responsible for the extended stable damage phase (Stage II) observed in rubberized asphalt specimens.

### 3.4. Decoupled Analysis of Fatigue Crack Propagation Kinetics and Service Life

For layered heterogeneous structures like rigid-flexible composite beams, conventional linear-elastic fracture mechanics parameters such as the nominal stress intensity factor range ∆K are no longer applicable once a crack propagates to the interlayer interface. The large mismatch in elastic modulus gives rise to mixed-mode fracture, crack-tip blunting and pronounced viscoelastic-plastic deformation in the asphalt layer. To achieve objective quantification of fatigue crack propagation resistance without the constraints of linear-elastic assumptions, the cyclic J-integral (∆J) approach was employed in this work. The irreversible energy dissipated per loading cycle (*w*_i_), obtained directly from macroscopic load–deflection hysteresis loops, was used to formulate a nonlinear equivalent stress intensity factor range (∆*K*_eq_). The value of ∆*K*_eq_ is expressed in Equation (4):

(4) $$∆K_{eq}=\sqrt{∆J\cdot E}$$

This energy-based equivalence approach inherently consolidates complex interfacial yielding, crack deflection, and Mode II shear sliding into a single equivalent fracture driving parameter. The quantitative relationship between the macroscopic fatigue crack growth rate (*d*a/*d*N) and ∆*K*_eq_ is well described by the modified Paris law (Equations (5) and (6)):

(5) $$\frac{da}{dN}=A{\left({∆K}_{eq}\right)}^{n}$$

(6) $$\log_{10}\left(\frac{\mathrm{da}}{\mathrm{dN}}\right)=\log_{10}A+n\log_{10}\left({∆K}_{eq}\right)$$

where *A* is the scaling regression coefficient, and *n* is the equivalent crack propagation resistance parameter for the composite interlayer system.

**Figure 9:**
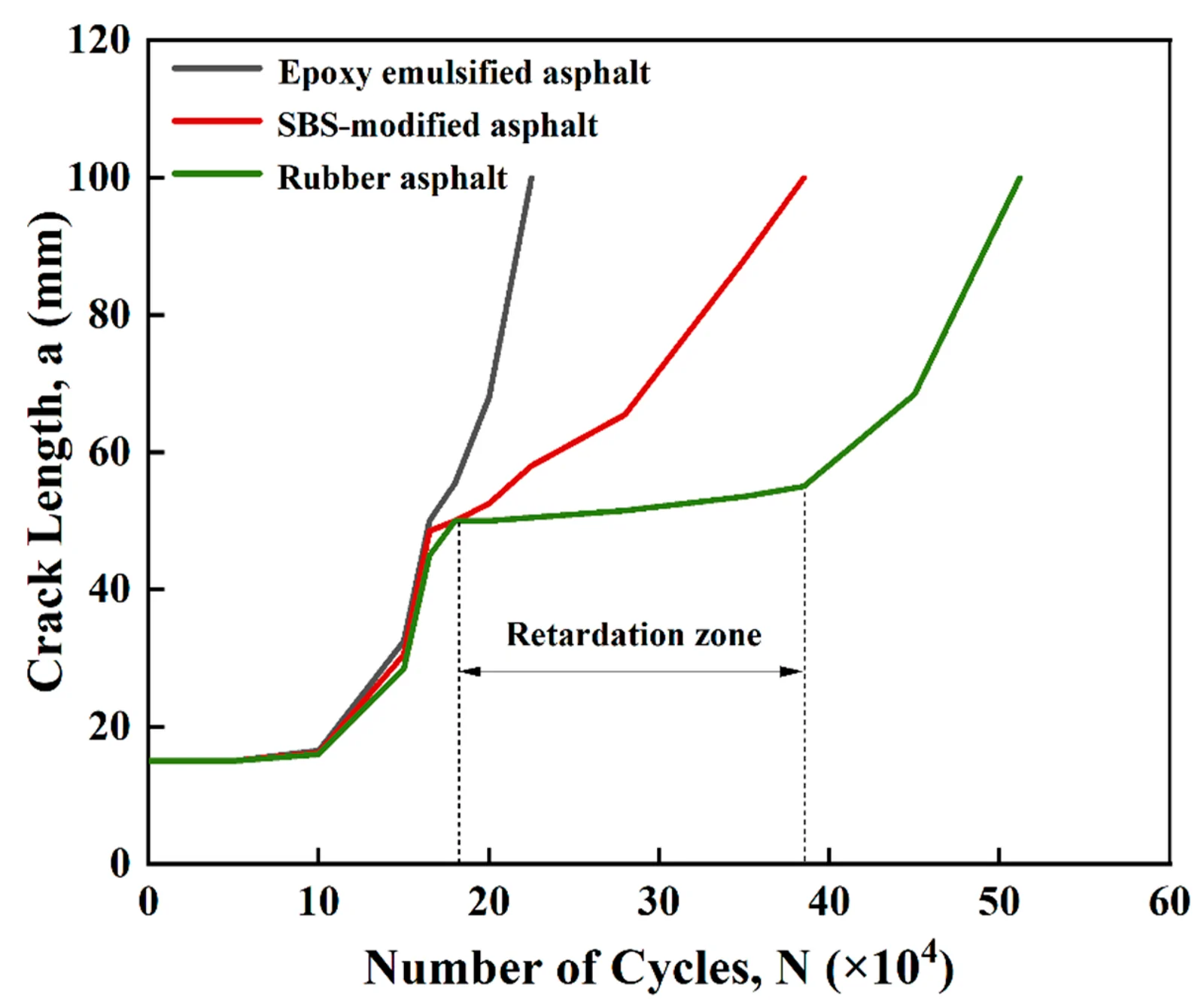
Evolution of crack length a with loading cycle number N.

Figure 9 illustrates the evolution of crack length a with loading cycles N, where the crack propagation process shows clear stage-wise characteristics. The composite beams adopted a 50 mm thick C40 concrete base course at the bottom, so the crack tip reaches the base-surface interface when a = 50 mm. When the crack propagates within the base course (a < 50 mm), the crack growth rates of the three groups are largely comparable, indicating that crack initiation and early propagation in the base course are barely influenced by the interfacial coating. Once the crack extends to the interface, all three coatings introduce a distinct crack growth deceleration zone. Specifically, the deceleration zone at the EEA interface is extremely narrow: the crack only stalls briefly before rapidly penetrating into the surface course. In contrast, the rubberized asphalt interface forms a broad deceleration zone spanning hundreds of thousands of loading cycles, exhibiting a much more pronounced crack retardation effect.

**Figure 10:**
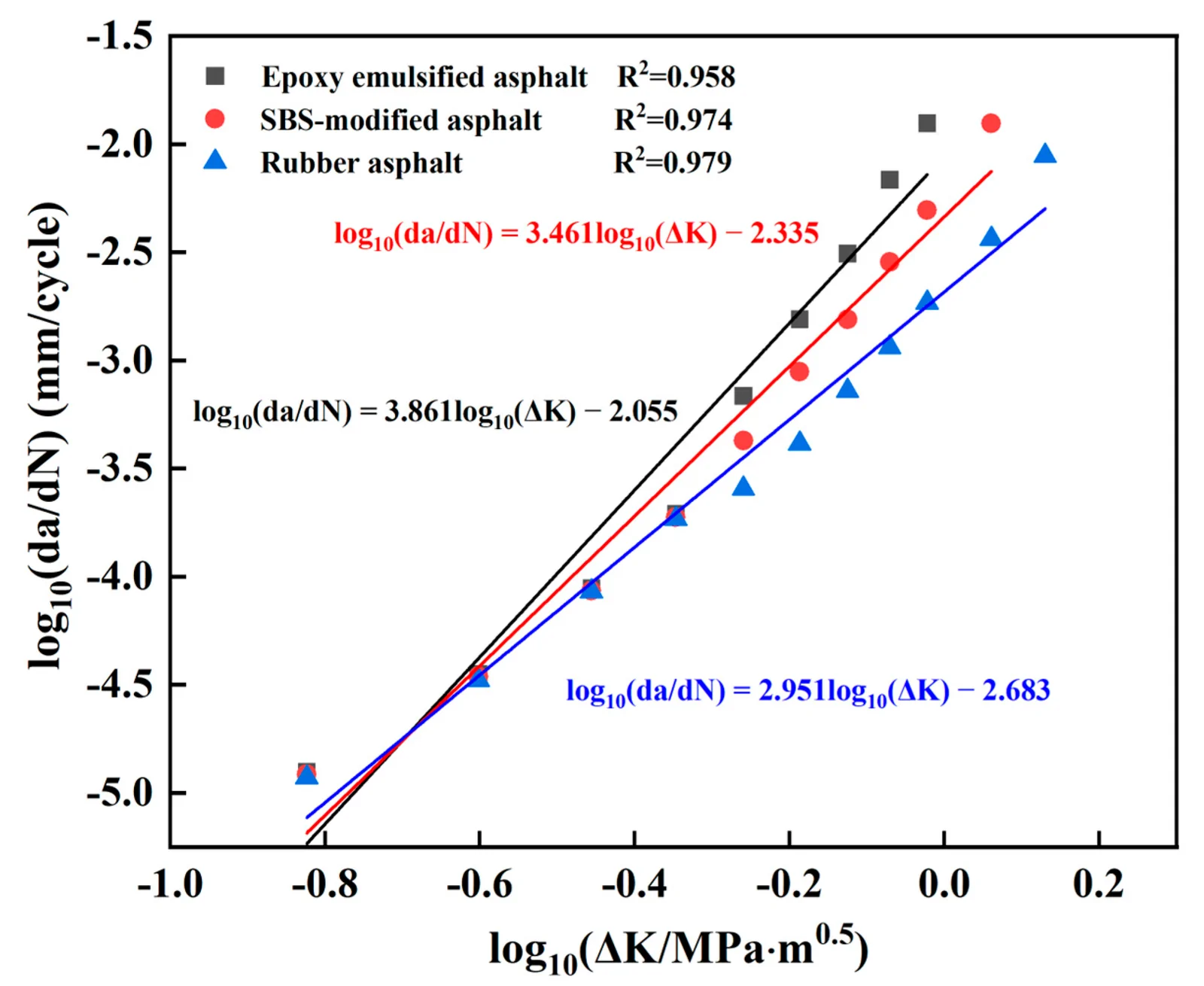
Paris law fitting of fatigue crack propagation rates for the three interfacial coatings.

As shown in Figure 10, linear regression of the post-interfacial crack growth data exhibits strong linear correlations (all R^2^ > 0.95). The fitted exponent *n* for the EEA interface is determined to be 3.861. This relatively high value indicates that once a crack penetrates through the rigid thermosetting EEA layer, the crack growth rate rises sharply with even small increments in the equivalent fracture driving force (∆*K*_eq_). By comparison, the corresponding *n* value for rubberized asphalt is the lowest, at 2.951, while SBS-modified asphalt yields an intermediate exponent of 3.461. Quantitatively, at a fixed equivalent driving force of ∆*K*_eq_ = 1.0 MPa∙m^0.5^, the extrapolated crack growth rate (*d*a/*d*N) of the EEA system is roughly one order of magnitude higher than that of the rubberized asphalt system. This energy-based comparison of crack growth kinetics further confirms that the large-deformation compatibility of rubberized asphalt confers the greatest resistance to unstable fatigue crack growth.

**Figure 11:**
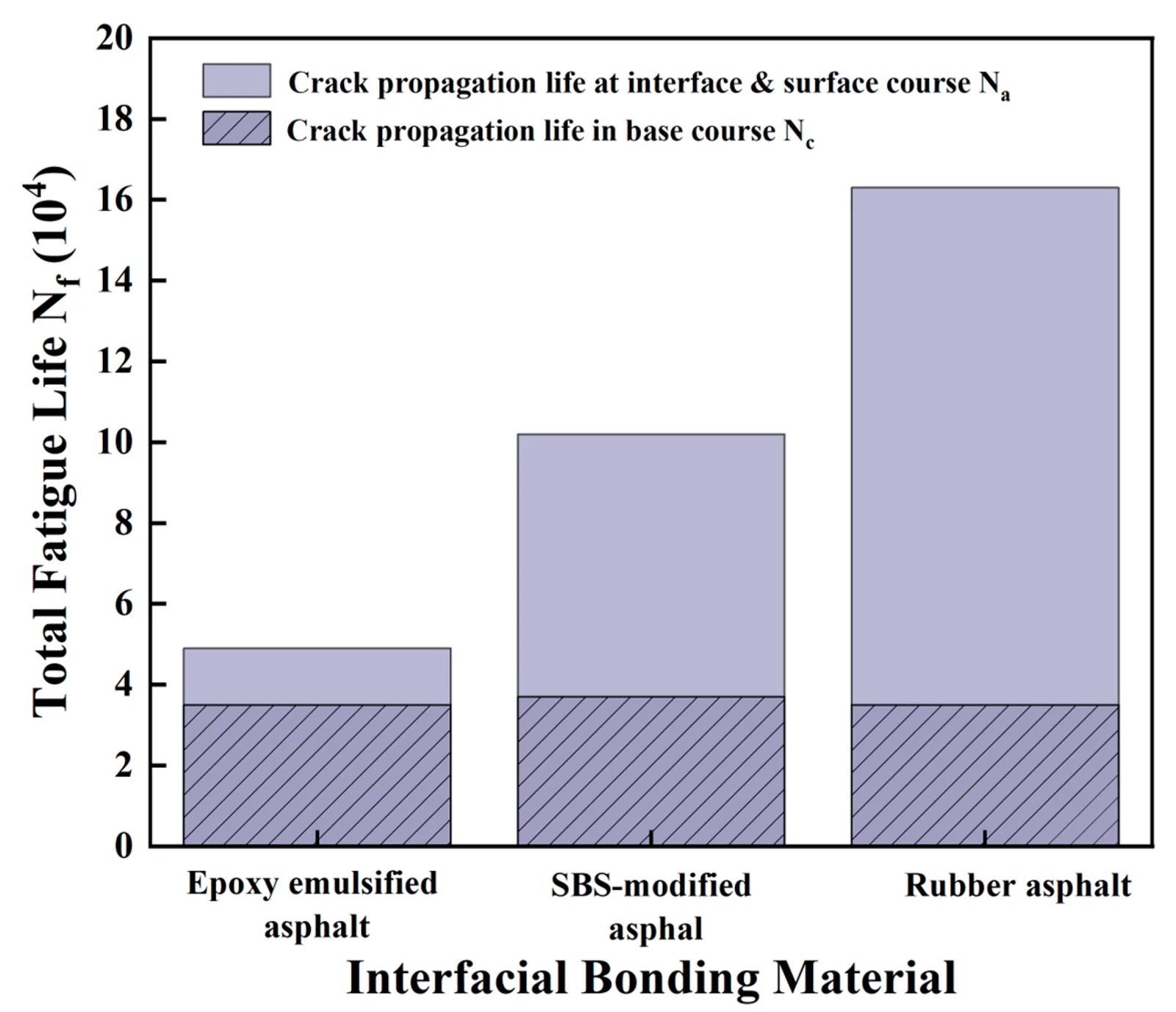
Decomposition of total fatigue life (N_c_ and N_a_) for composite beams with three interfacial coatings.

Figure 11 partitions the total fatigue life into two successive stages: the concrete base propagation stage (N_c_) and the interface-to-surface propagation stage (N_a_). One-way analysis of variance (ANOVA) of the N_c_ values across the three material groups returns *p* > 0.05, which statistically verifies that the crack propagation life within the concrete base course is invariant. As the C40 concrete base dominates the overall bending stiffness before the crack penetrates the interlayer, the 0.2–2.4 mm thick interlayer exerts a negligible effect on the crack driving force within the rigid concrete substrate. The superior overall fatigue performance of rubberized asphalt—with N_a_ exceeding 120,000 cycles-stems entirely from its flexible buffering effect during the post-interfacial propagation stage.

## 4. Conclusions

Based on static three-point bending tests and cyclic fatigue tests, this study comparatively investigated the mechanical degradation behavior and crack resistance mechanisms of three interfacial anti-cracking coatings: EEA, SBS-modified asphalt, and rubberized asphalt. The key scientific advance of this study is the construction of a two-stage decoupling framework for fatigue life (N_c_ and N_a_), coupled with a derived nonlinear cyclic J-integral (∆K_eq_) formulation. This analytical strategy breaks through the limitations of linear-elastic fracture mechanics in dealing with bimaterial interfaces with modulus mismatch, and achieves quantitative correlation between macroscopic crack growth retardation and mesoscale hysteretic energy dissipation. The main conclusions are drawn as follows:

(1) The three coatings differ notably in optimal application rate and static fracture behavior. Interfacial shear strength follows an inverted U-shaped trend with increasing application rate. Both EEA and SBS-modified asphalt achieve their peak shear strength at an application rate of 0.8 kg/m^2^, with values of 2.35 MPa and 1.48 MPa, respectively. Rubberized asphalt requires a higher application rate due to rubber particle swelling, reaching its peak strength of 0.98 MPa at 2.0 kg/m^2^. Static bending tests showed that EEA yielded the highest peak load of 12.6 kN but experienced rapid load loss after the peak, representing typical high-strength brittle fracture. Rubberized asphalt had the lowest peak load of 9.1 kN but maintained a residual bearing capacity above 2 kN at a mid-span deflection of 7 mm, indicating superior displacement ductility and deformation buffering performance. Quantitative sensitivity analysis corroborates the superior damage tolerance of rubberized asphalt. As the initial base-course notch depth increases from 10 mm to 20 mm, the material records a relative peak load reduction of just 25%—the lowest value among the three material groups—and retains a residual bearing capacity above 2 kN at a mid-span deflection of 7 mm.
(2) With respect to temperature dependence and crack propagation kinetics, the fatigue life of all examined interlayer systems attains its peak at 15 °C. This behavior arises from the temperature-dependent mechanical balance between low-temperature glassy embrittlement and high-temperature viscoelastic softening within the asphalt matrix. The absolute value of the stress sensitivity coefficient of rubberized asphalt was 5.278, markedly lower than those of EEA (6.860) and SBS-modified asphalt (5.817), indicating better adaptability to heavy load conditions. Decoupled life analysis based on the Paris law showed that the base course crack initiation life (N_c_) was approximately 35,000 cycles and was barely influenced by the interfacial coating material. Rubberized asphalt extended the crack propagation life (N_a_) across the interface and surface course to over 120,000 cycles with a longer crack growth deceleration stage, and its Paris law exponent *n* was 2.951, the lowest among the three coatings.
(3) The stiffness degradation profiles and load–deflection hysteresis responses confirm that high-modulus EEA primarily accumulates input mechanical work in the form of elastic strain energy. Over 81% of the initial stiffness is maintained through 80% of the fatigue service life, but this stored elastic energy triggers fast, unstable crack propagation as soon as the cohesive strength threshold is breached. By contrast, rubberized asphalt dissipates mechanical energy continuously through viscoelastic damping effects and rubber particle cavitation. Its total cumulative dissipated energy reaches approximately 6000 J, one order of magnitude greater than that of EEA, and the material undergoes a steady stiffness reduction, with 66.7% stiffness retention at N/N_f_ = 0.9. Accordingly, for heavy-traffic composite tunnel pavements and rehabilitated PCC pavements containing inherent joints and microcracks, priority should be given to high-damping, large-deformation interlayer materials (e.g., rubberized asphalt with a dosage of 2.0 kg/m^2^) in engineering design, instead of focusing solely on high static interfacial shear strength.
(4) The modified three-point bending test adequately reproduces the coupled tensile–shear stresses intrinsic to reflective cracking. However, this static laboratory test configuration does not account for the continuous elastic subgrade support (Winkler foundation) and principal stress rotation induced by rolling wheel loads in actual pavement structures. Further investigation using high-resolution X-ray computed tomography (CT) is required to directly observe and validate the postulated internal micropore deformation mechanisms. Additionally, accelerated pavement testing (APT) is recommended to calibrate the proposed two-stage fatigue life decomposition approach under full-scale mixed-mode traffic loading conditions.

## Data Availability

The raw data supporting the conclusions of this article will be made available by the authors on request.

## References

[1] Li S., Liu Z.H., Li Y.Z. (2012). Influence of Structure Layer Thickness of CRC+AC Composite Pavement on Temperature Effect and Rutting Deformation. China J. Highw. Transp..

[2] Shi Z., Yue J., Xu L., Wang X. (2022). Peridynamics for fracture analysis of reflective cracks in semi-rigid base asphalt pavement. Appl. Sci..

[3] Nagavanshi A.K. (2026). Mechanisms of Fracture, Cracking and Material-Level Mitigations in Asphalt Mixes and Asphalt Layers: A Critical Review. Crack. Mater.-Level Mitig. Asph. Mix. Asph. Layers A Crit. Rev..

[4] Fang N., Wang X., Chang H., Yu K. (2025). Design approach for composite pavement structure incorporating reflective crack considerations. Appl. Sci..

[5] Yan C., Wei Y. (2023). Dynamic response analysis of JPCP with different roughness levels under moving axle load using a numerical methodology. Appl. Sci..

[6] Yu J., Fan K., Feng Z., Zou G., Zhang Y., Yu H. (2026). A comprehensive review on reflective cracking in asphalt pavements: Formation, evaluation, and treatment. Road Mater. Pavement Des..

[7] Li P., Xiao X., Tian S., Liu J., Peng W., Wang B., Liu S. (2023). Gradation influence on crack resistance of stress-absorbing membrane interlayer. Appl. Sci..

[8] Kong L., Su S., Wang Z., Wu P., Zhang Y., Chen Z., Ren D., Ai C. (2024). Microscale mechanism and key factors of waterborne epoxy resin emulsified asphalt enhancing interlayer bonding performance and shear resistance of bridge deck pavement. Constr. Build. Mater..

[9] Dai Y., Zhang H.L. (2024). Interlayer Bonding Behavior of Waterborne Epoxy Emulsified Asphalt Ultra-thin Wearing Course. J. Highw. Transp. Res. Dev..

[10] Li X., Shen J., Ling T., Du H. (2022). Multi-index evaluation for anticracking performance of epoxy porous asphalt mixtures based on overlay test. J. Mater. Civ. Eng..

[11] Idris I.I., Sadek H., Hassan M. (2020). State-of-the-art review of the evaluation of asphalt mixtures’ resistance to reflective cracking in laboratory. J. Mater. Civ. Eng..

[12] Pan R., Li Y. (2020). Effect of warm mix rubber modified asphalt mixture as stress absorbing layer on anti-crack performance in cold region. Constr. Build. Mater..

[13] Xu G., Yao Y., Wu M., Zhao Y. (2023). Molecular simulation and experimental analysis on co-aging behaviors of SBS modifier and asphalt in SBS-modified asphalt. Mol. Simul..

[14] Yu H., Bai X., Qian G., Wei H., Gong X., Jin J., Li Z. (2019). Impact of ultraviolet radiation on the aging properties of SBS-modified asphalt binders. Polymers.

[15] Chen H., Qiu H., Wu Y., Kuang D., Xing M. (2022). Aging resistance of SBS–SBR composite modified asphalt materials for high altitude and cold regions. Mater. Struct..

[16] (2011). Specifications for Design of Highway Cement Concrete Pavement.

[17] (2004). Technical Specification for Construction of Highway Asphalt Pavement.

[18] (2005). Test Methods of Aggregate for Highway Engineering.

[19] Ministry of Transport of the People’s Republic of China (2020). Testing Methods of Cement and Concrete for Highway Engineering.

[20] (2019). Specifications for Design and Construction of Pavement on Highway Steel Deck Bridge.

[21] Zhu Z., Al-Qadi I.L. (2023). Crack detection of asphalt concrete using combined fracture mechanics and digital image correlation. J. Transp. Eng. Part B Pavements.

[22] Li Q., Wu H., Song W., Chen X., Wang M., Sun Y., Liao H. (2025). Characterization on thermal fatigue damage in asphalt pavement under cyclic temperature variations. Constr. Build. Mater..

[23] Xie J., Xu H., Huang T. (2024). Simulation study on crack extension law of asphalt pavement under temperature effect. Front. Built Environ..

[24] Wang Y., Wei J., Chi F., Sun Y. (2025). Evaluation of fatigue behavior in asphalt mixtures using bidirectional splitting and indirect tensile testing. Matéria.

[25] Cheng H., Sun L., Wang Y., Chen X. (2021). Effects of actual loading waveforms on the fatigue behaviours of asphalt mixtures. Int. J. Fatigue.

[26] Diakhaté M., Millien A., Petit C., Phelipot-Mardelé A., Pouteau B. (2011). Experimental investigation of tack coat fatigue performance: Towards an improved lifetime assessment of pavement structure interfaces. Constr. Build. Mater..

[27] Lv S., Wang Z., Zhu X., Yuan J., Peng X. (2023). Research on strength and fatigue properties of asphalt mixture with different gradation curves. Constr. Build. Mater..

[28] Liang Y., Ma S., Zhang Y. (2024). Interlayer performance, viscoelastic performance, and road performance based on high-performance asphalt composite structures. Buildings.

[29] Abualia A., Akentuna M., Mohammad L.N., Cooper S.B., Cooper S.B. (2024). Improving Asphalt Binder Durability Using Sustainable Materials: A Rheological and Chemical Analysis of Polymer-, Rubber-, and Epoxy-Modified Asphalt Binders. Sustainability.

[30] Gao Y., Geng D., Huang X., Cao R., Li G. (2018). Prediction of remaining service life of asphalt pavement using dissipated energy method. J. Transp. Eng. Part B Pavements.

[31] Xie P., Wang H. (2022). Finite element analysis of thermal-induced reflective cracking in composite pavement with mitigation strategies. Eng. Fract. Mech..

[32] Al-Jarazi R., Rahman A., Ai C., Al-Huda Z., Abdelhliem B.L.E. (2024). Interface bonding strength between asphalt pavement layers under mixed shear-tensile mode: Laboratory evaluation and modeling predictions. J. Mater. Civ. Eng..

[33] Yang K., Li R., Yu Y., Pei J. (2021). Unified laboratorial evaluation of interlayer bond property in asphalt pavements based on strength parameters. Constr. Build. Mater..

[34] Sun R., Li J., Kong L. (2026). Interfacial Shear Fatigue and Damage Evolution of Epoxy-Emulsified Asphalt Bond Coats Under Coupled Effects of Temperature, Loading Frequency and Stress Level. Coatings.

[35] Kumar A., Choudhary R. (2025). Evaluating rheological and mechanical properties of asphalt binders and mixes with waste EPDM rubber and Pyro-oil composites. J. Mater. Civ. Eng..

[36] Yang S., Li R., Zhu H., Qin Y., Huang C. (2024). Review of the state-of-the-art techniques for enhancing the toughness of thermosetting epoxy asphalt. Constr. Build. Mater..

[37] Mahmoudi Y., Mangiafico S., Sauzéat C., Di Benedetto H., Pouget S., Faure J.-P. (2023). Energy Dissipation of Bituminous Mixtures with Crumb Rubber Added by Dry Process: Laboratory Tests and Numerical Simulation. J. Test. Eval..

[38] Li P., Peng W., Tian S., Liu Z., Liu J., Liu S. (2023). Comprehensive laboratory evaluation of crack resistance for an asphalt rubber stress-absorbing membrane interlayer (AR-SAMI). Sustainability.

